# ERC-BiP Functional Protein Pathway for Assessing Endoplasmic Reticulum Stress Induced by SARS-CoV-2 Replication after Cell Invasion

**DOI:** 10.1155/2023/7253779

**Published:** 2023-10-09

**Authors:** Mingshan Xue, Zhiwei Lin, Teng Zhang, Zhangkai J. Cheng, Runpei Lin, Baojun Guo, Yifeng Zeng, Fengyu Hu, Feng Li, Peiyan Zheng, Huimin Huang, Ning Li, Qi Zhao, Baoqing Sun, Xiaoping Tang

**Affiliations:** ^1^Guangzhou Eighth People's Hospital, Guangzhou Medical University, Guangzhou 510060, China; ^2^Guangzhou Laboratory, XingDaoHuanBei Road, Guangzhou International Bio-Island, Guangzhou 510005, Guangdong Province, China; ^3^National Center for Respiratory Medicine, The First Affiliated Hospital of Guangzhou Medical University, National Clinical Research Center for Respiratory Disease, State Key Laboratory of Respiratory Disease, Guangzhou Institute of Respiratory Health, Guangzhou 510120, China; ^4^Cancer Centre, Institute of Translational Medicine, Faculty of Health Sciences, University of Macau, Taipa, Macau, China; ^5^MoE Frontiers Science Center for Precision Oncology, University of Macau, Taipa, Macau, China; ^6^School of Medicine, Henan University, Kaifeng 475000, Henan, China

## Abstract

**Background:**

SARS-CoV-2 induces apoptosis and amplifies the immune response by continuously stressing the endoplasmic reticulum (ER) after invading cells. This study aimed to establish a protein-metabolic pathway associated with ER dysfunction based on the invasion mechanism of SARS-CoV-2.

**Methods:**

This study included 17 healthy people and 46 COVID-19 patients, including 38 mild patients and 8 severe patients. Proteomics and metabolomics were measured in the patient plasma collected at admission and one week after admission. The patients were further divided into the aggravation and remission groups based on disease progression within one week of admission.

**Results:**

Cross-sectional comparison showed that endoplasmic reticulum molecular chaperone-binding immunoglobulin protein (ERC-BiP), angiotensinogen (AGT), ceramide acid (Cer), and C-reactive protein (CRP) levels were significantly increased in COVID-19 patients, while the sphingomyelin (SM) level was significantly decreased (*P*  <  0.05). In addition, longitudinal comparative analysis found that the temporal fold changes of ERC-BiP, AGT, Cer, CRP, and SM were significantly different between the patients in the aggravation and remission groups (*P*  <  0.05). ERC-BiP, AGT, and Cer levels were significantly increased in aggravation patients, while SM was significantly decreased (*P*  <  0.05). Meanwhile, ERC-BiP was significantly correlated with AGT (*r* = 0.439; *P*  <  0.001).

**Conclusions:**

ERC-BiP can be used as a core index to reflect the degree of ER stress in COVID-19 patients, which is of great value for evaluating the functional state of cells. A functional pathway for AGT/ERC-BiP/glycolysis can directly assess the activation of unfolded protein reactions. The ERC-BiP pathway is closer to the intracellular replication pathway of SARS-CoV-2 and may help in the development of predictive protocols for COVID-19 exacerbation.

## 1. Introduction

The surface S protein of the SARS-CoV-2 virus invades the cytoplasm by binding to the host cell receptor angiotensin-converting enzyme 2 (ACE2) [1]. The virus then replicates in the endoplasmic reticulum (ER) [1]. The properties of rapid membrane fusion promote SARS-CoV-2 replication, propagation, and migration along the respiratory tract, inducing innate immune and inflammatory responses [2, 3]. Respiratory symptoms are the main clinical manifestation of COVID-19 patients [2]. There is pathological evidence of bilateral diffuse alveolar injury, hyaline membrane formation, fibrin deposition, and exfoliation of pulmonary epithelial cells in COVID-19 patients [3]. Mild COVID-19 patients may experience none or only respiratory symptoms, while severe patients may develop acute respiratory distress syndrome (ARDS) or multiple organ dysfunction syndrome (MODS) [4]. As a result, the evaluation of COVID-19 exacerbation has attracted much interest.

The increased risk of COVID-19 is closely related to the viral burden and inflammatory storm. ER is one of the largest organelles in the cell where protein folding and secretion, membrane synthesis, and lipid synthesis occur. It is also one of the largest assembly regions for SARS-CoV-2, providing the membrane structure requisite for virus replication and release [5, 6]. ER controls lipid metabolism, calcium storage, and protein folding balance [7, 8]. ER membrane is in a dynamic equilibrium state. ER also acts as a regulator, detecting and repairing incorrectly translated proteins [9]. Direct injury and indirect inflammation induced by SARS-CoV-2 disrupt the stable protein synthesis environment of ER [9]. The accumulation of misfolded proteins triggers unfolded protein reaction (UPR), causing ER stress [9]. A persistent stress signal triggers autophagy, leading to apoptosis and immune response amplification [6].

Therefore, the evaluation of ER functions in COVID-19 patients could provide insights into the mechanism of SARS-CoV-2-induced cellular dysfunction. Heat shock protein (HSP70, also named BiP) has a molecular weight of 70 kD. HSP70 is a ubiquitous molecular chaperone involved in a variety of cellular functions, protecting cells from the effects of various viral invasive stresses [10]. However, it has also been suggested that Hsp70 family proteins interact directly with viral polymerases to enhance viral replication or that they may promote the formation of viral replication complexes and/or maintain the stability of complex proteins [11]. As an important macromolecule assisting in protein folding and assembly, BiP plays different functions in different parts. In ER, BiP can regulate the activity and transport of proteins. For this kind of Hsp70 (BiP), the unified name is ERC-BiP. Endoplasmic reticulum molecular chaperone-binding immunoglobulin protein (ERC-BiP), which regulates protein folding, plays a key role in ER homeostasis [12]. Furthermore, ERC-BiP maintains the ER permeability barrier during protein translocation and retrograde translocation for misfolded proteins [13]. However, proteasomes can degrade ERC-BiP, thus promoting ER calcium storage and UPR activation by sensing stress [13]. Therefore, ERC-BiP can be used to evaluate the function of ER. The formation of a protein network may aid in assessing homeostasis deviation caused by SARS-CoV-2. In this study, ERC-BiP was selected to evaluate the state of ER stress induced by SARS-CoV-2 and establish a systematic functional protein pathway, which may provide insights into the mechanism of COVID-19 disease progression and help in the development of predictive protocols for COVID-19 exacerbation.

## 2. Materials and Methods

### 2.1. Inclusion Criteria

A total of 46 COVID-19 patients were recruited from the Eighth Hospital of Guangzhou Medical University. The patients were divided into the mild group (*n* = 38) (patients with mild clinical symptoms and mild pneumonia on imaging) and the severe group (*n* = 8) (respiratory rate ≥30 breaths/min, SaO_2_ ≤93%, and PaO_2_/FiO_2_ ≤300 mmHg at the resting state, imaging indicating a lung exudate lesion area progressing by more than 50% within 24-48 hours, and the necessity for mechanical ventilation, etc.) based on the ninth edition of the COVID-19 guidelines of the National Health Commission of China.

Clinical data and blood samples of patients were collected at admission and one week after admission [14]. The patients were divided into two groups based on their conditions at the two time points, except for 5 patients without longitudinal samples, as follows: the aggravation group (*n* = 29) (patients with significant respiratory symptom exacerbation, increased area of pulmonary imaging lesions, and decreased oxygenation index after admission) and the remission group (*n* = 12) (patients with reduced symptoms, decreased pulmonary lesions, or improved clinical indices). The evaluation was conducted by three researchers, two laboratory physicians, and two clinicians based on the patients' examination results or symptoms. Moreover, 17 healthy controls were included.

### 2.2. Plasma Collection

Blood samples were collected from subjects early in the morning. The samples were centrifuged at 3000 rpm and room temperature for 10 min within two hours. The samples were then stored in isolation at −80°C. Each sample was aspirated with 50 *μ*l of plasma dispensed for further analysis [14].

### 2.3. Target Proteomics Analysis

Target proteomics analyses were conducted using ultraperformance liquid chromatography (UPLC, ExionLC AD, Shanghai, China. https://sciex.com.cn/) and tandem mass spectrometry (MS/MS. QTRAP®, https://sciex.com.cn/). Through the selective detection of specific peptide sequences or target peptide segments, such as those undergoing post-translational modifications, it is possible to achieve targeted relative quantification of ERC-BiP. This approach makes use of the selective detection capability of a quadrupole mass analyzer in the first-stage mass spectrometry (Q1) to accurately identify the precursor ion information of the target peptide segment. Subsequently, the peptide is fragmented in the collision-induced dissociation (CID) cell, and the resulting fragmented ions are then analyzed in a high-resolution, high-accuracy mass analyzer. By employing this comprehensive methodology, ERC-BiP can be analyzed with precision and specificity in complex samples, without the reliance on ERC-BiP antibodies. Notably, this approach effectively eliminates background interference and false positives, thereby significantly enhancing the sensitivity of ERC-BiP detection in complex backgrounds.

### 2.4. Metabolomics Detection Techniques

The study employed state-of-the-art liquid chromatography-mass spectrometry (LC-MS) techniques (UPLC, ExionLC AD, Shanghai, China. https://sciex.com.cn/) to perform targeted metabolomics. The sample extracts were subjected to chromatographic separation using a high-performance liquid chromatography system. The separated metabolites were then ionized and introduced into a mass spectrometer for detection.

For the detection and quantification of specific metabolites, we utilized multiple reaction monitoring (MRM) in the mass spectrometer. MRM enables the precise selection of parent and daughter ion pairs corresponding to the metabolites of interest. This targeted detection strategy significantly enhances the sensitivity and specificity of metabolite analysis, enabling the accurate quantification of low-abundance metabolites in complex biological samples. The study primarily focused on lipid metabolites and energy cycle-related metabolites.

### 2.5. Standardization of Omics Data

During the preprocessing of serum samples, 5 *μ*l of internal standards, HETE-d8 and Phe-2, were added to each sample. The peak area of each metabolite (Area i) was then divided by the peak area of the corresponding internal standard (area internal) in the same sample, resulting in a relative abundance value. Since an equal amount of internal standard was added to each sample, this calibration helps to normalize the metabolite levels to the time of internal standard addition. In PRM experiments based on mass spectrometry, the fragmentary signal entering the detector in the actual sample is collected by mass spectrometry and then the relative signal strength of the target protein is compared between different groups.

### 2.6. Statistical Analysis

Continuous variables were expressed as the median (interquartile range (IQR)), while categorical variables were expressed as the frequency. The Mann–Whitney–Wilcoxon rank-sum test (two groups) or the Kruskal–Wallis test (three or more groups) was used to assess differences in continuous variables. The Chi-square test (two groups) or Fisher's exact test (three or more groups) was used to compare the categorical data. Spearman's correlation coefficient was calculated to explore the correlation between different metabolites and proteins. Metabolite and protein levels were separately measured at the two time points. The change in metabolite levels was determined by finding the difference between the values of the second measurement and the first measurement. Partial least squares-discriminant analysis (PLS-DA) was used to screen metabolites. *P*  <  0.05 was considered statistically significant. R software version 4.0.0 (R Core Team) was used for all statistical analyses.

## 3. Results

### 3.1. Characteristics of the Participants

Lymphocytes (LYM and LYM%), monocytes (MONO and MONO%), and basophils (BASO and BASO%) were significantly decreased in COVID-19 patients than those in healthy controls (*P*  <  0.001) (Table 1). Moreover, human serum amyloid A (SAA) levels were higher in COVID-19 patients (normal range: 0–10 ng/ml). However, most indices were not significantly different between patients with disease aggravation and remission (Supplementary Table 1).

### 3.2. Horizontal Comparison of Protein and Metabolite Levels in COVID-19 Patients

**Figure 1:**
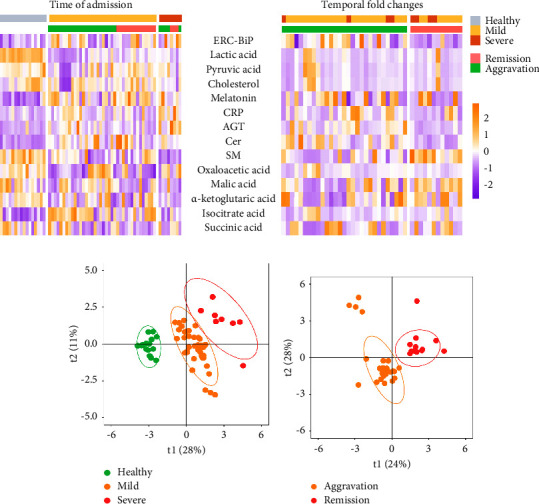
(a) Heatmap showing the levels of different proteins/metabolites at admission (left) and the difference between the second measurement and the first measurement. (b) The PLS-DA scores plot showing the metabolites and proteins in the healthy control, mild patients, and severe patients (*R*^2^ = 0.779, *Q*^2^ = 0.706, and *P*  <  0.05). (c) The PLS-DA scores plot showing the differences of metabolites and proteins in the aggravation group, constant group, and remission group (*R*^2^ = 0.993, *Q*^2^ = 0.978, and *P*  <  0.05). SM: sphingomyelin; CRP: C-reactive protein; AGT: angiotensinogen; ERC-BiP: endoplasmic reticulum companion-BiP; Cer: ceramide acid.

A total of 14 ERC-BiP-associated metabolites and proteins were measured in healthy controls, mild, and severe COVID-19 patients. The distributions of metabolites and proteins are shown using heatmap in Figure 1(a). PLS-DA analysis showed that the significant separation of metabolite and protein levels among the three groups in Figure 1(b). The significantly differences and fold change values between three groups are shown in Table 2. The variables of importance projection (VIP) of all metabolites or proteins were calculated by PLS-DA (Supplementary Figure 1). The results showed that ERC-BiP levels were significantly higher in COVID-19 patients than in healthy controls (log2(FC) = 0.615, *P* = 0.035). Moreover, ERC-BiP levels were significantly higher in severe patients than in mild patients (log2 (FC) = 0.995 and *P* = 0.018). Melatonin, angiotensinogen (AGT), CRP, and ceramide acid (Cer) levels were upregulated in COVID-19 patients (log2 (FC) = 0.486, 1.191, 2.002, and 1.712; *P*  <  0.05). However, lactate acid, pyruvate acid, and sphingomyelin (SM) levels were downregulated in COVID-19 patients (log2 (FC) = −0.946, −0.361, and −0.946; *P*  <  0.05).

### 3.3. Horizontal Comparison of Protein and Metabolite Levels in COVID-19 Patients

The patients were divided into the aggravation group (*n* = 29) and the remission group (*n* = 12) based on the changes in their condition after one week of admission to assess the disease progression. The temporal fold change was defined as the fold change between the second measurement of metabolites and proteins and the first measurement, i.e., the second measurement value/the first measurement value. The distributions of the temporal fold changes are shown using heatmap in Figure 1(a). PLS-DA analysis showed significant separation of the temporal differences among the aggravation and remission groups in Figure 1(c). The detailed values of the temporal fold changes in each group are shown in Table 3. The temporal fold changes of ERC-BiP, CRP, AGT, Cer, and SM were significantly different between aggravation group and remission group (*P*  <  0.05). However, the temporal fold changes of melatonin were not significantly different among these groups.

**Figure 2:**
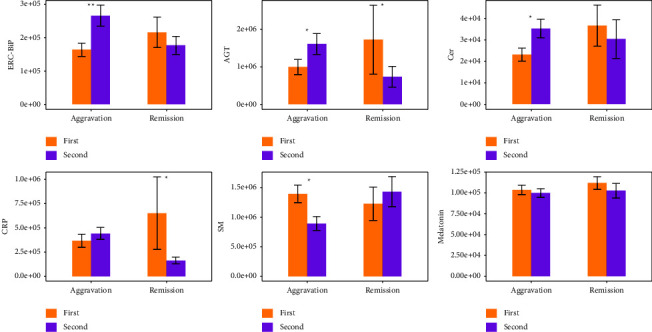
Longitudinal time-point comparison of COVID-19 patients at various progression stages. The asterisks ^*∗*^ and ^*∗∗*^ represent *P*  <  0.05 and *P*  <  0.01, respectively.

The longitudinal temporal changes in metabolites and proteins in each group are expressed in histograms in Figure 2. ERC-BiP, AGT, and Cer levels were significantly increased in the aggravation group after one week, while SM was significantly decreased (*P*  <  0.05). Moreover, CRP was significantly decreased in the remission group (*P*  <  0.05), while SM was slightly elevated. ERC-BiP, AGT, and Cer levels were also slightly elevated in the remission group (*P*  >  0.05). There were no significantly differences of melatonin in either aggravation group or remission group.

**Figure 3:**
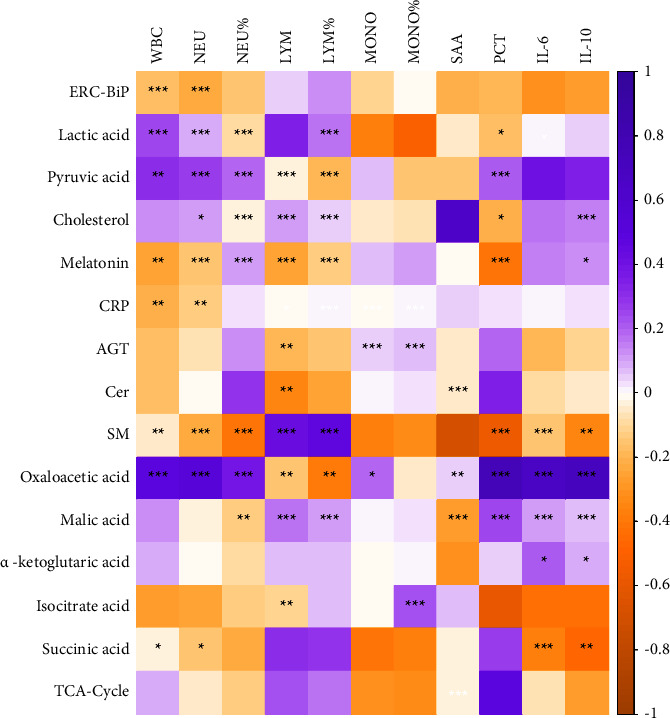
Correlation analysis between functional pathway indicators of ERC-BiP and clinical indicators. The asterisks ^*∗*^, ^*∗∗*^, and ^*∗∗∗*^ represent *P*  <  0.05, *P*  <  0.01, and *P*  <  0.001, respectively.

The correlations between clinical indicators and metabolites/proteins are calculated in Figure 3. The detailed values of correlation coefficients and *P* values are shown in Supplementary Table 2. The correlation analyst showed that the ERC-BiP was not significantly correlated with the immune cell count (WBC, NEU, LYM, and MONO) and inflammatory markers (SAA and PCT). However, ERC-BiP was significantly positively correlated with AGT (*r* = 0.439; *P*  <  0.001). ERC-BiP was also significantly correlated with Cer and lactate acid (*r* = 0.534 and *P*  <  0.001;*r* = 0.3528 and *P* = 0.005).

## 4. Discussion

In this study, lymphocytes of COVID-19 patients were significantly decreased. Moreover, ERC-BiP, which reflects the ER function, was significantly different between patients and healthy subjects and was significantly increased in severe patients. AGT, which mediates viral transmembrane action, was also associated with disease exacerbation. In COVID-19 patients, the equilibrium between anaerobic and aerobic circulation, the major pathway for protein folding in ER, was significantly altered. Furthermore, the activity of the TCA cycle (standardized population) and lactic acid accumulation was decreased in COVID-19 patients. As a result, significant inflammatory factor release syndrome occurred in severe patients, as shown by the significantly increased IL-6, CRP, and SAA levels.

### 4.1. ERC-BiP Reflects ER Stress and Cell Pyroptosis

The ERC-BiP levels in COVID-19 patients were significantly higher than those in healthy controls, while those in the severe group were significantly higher than those in the mild group. Moreover, longitudinal comparative analysis found that the temporal differences of ERC-BiP were significantly different between the patients in the aggravation and remission groups, and the ERC-BiP levels were significantly elevated in aggravation patients.

Proteins misfold during translation under normal conditions. Therefore, abnormal proteins should be timely removed to maintain intracellular homeostasis through ERC-BiP [12]. SARS-CoV-2 affects the normal function of ER, resulting in the emergence of several abnormally folded proteins, which then activate the protective stress state of the ER. Stress is mainly characterized by UPR [9]. UPR can promote apoptosis, angiogenesis, autophagy, innate immunity, and proinflammatory effects [6]. Therefore, rapid protein folding is crucial for the survival of ER-stressed cells [15]. UPR can inhibit protein translation, thus reducing the ER load. However, the upregulation of misfolded protein clearance factors restores ER function [16]. Therefore, the SARS-CoV-2-induced stress signal activates the massive release of ERC-BiP to recognize incomplete folding of glycosylated proteins in response to ER stress [9, 13], thus preventing the transformation of stress effect from protection to injury. Therefore, ERC-BiP can inhibit the intracellular abnormalities induced by SARS-CoV-2 replication after invasion, thereby delaying the risk of systemic inflammation caused by scoria following decompensated accumulation of abnormal proteins.

### 4.2. ERC-BiP Reflects ER Stress and Cell Pyroptosis

Angiotensinogen (AGT) and melatonin are key proteins affecting ER stress. In this study, AGT levels were significantly increased in COVID-19 patients and were significantly correlated with ERC-BiP. Melatonin levels were significantly increased in COVID-19 patients. However, melatonin levels were not significantly different between mild and severe patients.

AGT plays a crucial role in SARS-CoV-2 binding to cell surface sites. The ACE2 transmembrane protein receptor is the binding site of S-spike protein on the SARS-CoV-2 coat [15, 16]. Free ACE2 (sACE2) levels are elevated in COVID-19 patients, indicating its mediating function in SARS-CoV-2 invasion progression [17]. Therefore, COVID-19 infection is closely related to the renin-angiotensin system (RAS). AGT is the rate-limiting substrate of RAS. Cafiero et al. showed that AGT enhances SARS-CoV-2 binding to cells, tissues, and organs [18]. AGT is upstream of ERC-BiP and acts as a “valve” regulating ER function. ACE2 degrades AGT. The expression of ACE2 on the membrane surface decreases after SARS-CoV-2 infection [19]. Therefore, the decreased AGT conversion rate may increase AGT levels. The AGT-SACE2 system is associated with susceptibility to COVID-19 [20]. Elevated AGT levels may result in overactivation of the downstream Ang II/AT1R axis [21], thus increasing the risk of MODs and ARDS. However, it is unknown whether the increased AGT level is beneficial or harmful. Wang et al. [17], Hatmal et al. [22], and Cui et al. [23] have extensively studied the RAS pathway. In this study, the expression level of ACE2 was associated with COVID-19 progression. AGT can also control the replication process of the virus in ER. Melatonin acts as another “valve” controlling stress intensity. Melatonin has antioxidant and anti-inflammatory effects. It can affect the UPR pathway and reduce ER stress intensity [2, 9]. In this study, melatonin levels were significantly higher in COVID-19 patients than in healthy people. However, the longitudinal time difference analysis showed that melatonin secretion did not have a linear trend, indicating that melatonin release is not associated with the aggravated ER stress state [24–26]. The infection process requires cooperation between the virus and the host cell [27]. ERC-BiP does not independently play a protective role since substances upstream and downstream also interact with each other. ERC-BiP has both promotive and inhibitory effects, and the overall function of pathways is often diversified. Although melatonin was not correlated with AGT, external complementary intervention can inhibit ER stress and reduce the adverse effects of abnormal protein accumulation. Many studies have also shown that melatonin supplementation has a clinical value in COVID-19 treatment [28, 29].

### 4.3. Functional Evaluation of ER Synthesis and Protein Release

Cer-SM (ceramide-sphingomyelin), a metabolic pathway produced in ER and closely associated with cell pyrosis, was used to further explore the abnormalities of ER function in COVID-19 patients and the exact role of ERC-BiP. The two-protein pathway can be used to evaluate the impact of COVID-19 on ER function and the inflammatory state of the body. Clarke et al. [30] indicated that sphingolipids can affect the physical properties of membranes. ER induces membrane rearrangement and self-fusion into two-membrane-vesicle-structured autophagosomes under normal conditions to remove cell debris or pathogens. The balance of Cer-SM sphingolipid metabolism can maintain the dynamic equilibrium of the ER membrane. However, SARS-CoV-2 inhibits autophagosome and lysosome fusion and the cell clearance mechanism [24]. In this study, the Cer-SM pathway analysis showed that increased Cer and SM hydrolysis could lead to the stagnation of the cell cycle. Moreover, the amplification of stress signal can lead to pyroptosis [31]. Although Cer is widely distributed in the body, omics technology cannot accurately detect the Cer level in ER. However, the increased Cer level negatively affected the alleviating effect of UPR on abnormal protein folding. Therefore, ER can maintain its internal homeostasis by enhancing ERC-BiP even after the invasion of SARS-CoV-2 since ERC-BiP is significantly correlated with Cer. Although ER cannot effectively prevent the replication of the virus, it has a certain inhibitory effect on the activation of inflammation.

### 4.4. Energy Supply

The overall level of the TCA cycle decreases in COVID-19 patients while the lactic acid level increases. Protein folding and repair are energy-consuming processes whether ER is in the infected or defense states. SARS-CoV-2 invades the airway epithelium and triggers respiratory symptoms. This leads to hypoxia which inhibits the TCA activity, leading to the Warburg effect [32]. Pyruvate converts to lactic acid and accumulates in the cell. Lactic acidosis synergistically activates UPR and inflammatory response [33], thus affecting the ER membrane activity and protein folding and repair. Furthermore, the correlation between lactic acid and ERC-BiP indicates that energy supply disturbance affects the ER function.

**Figure 4:**
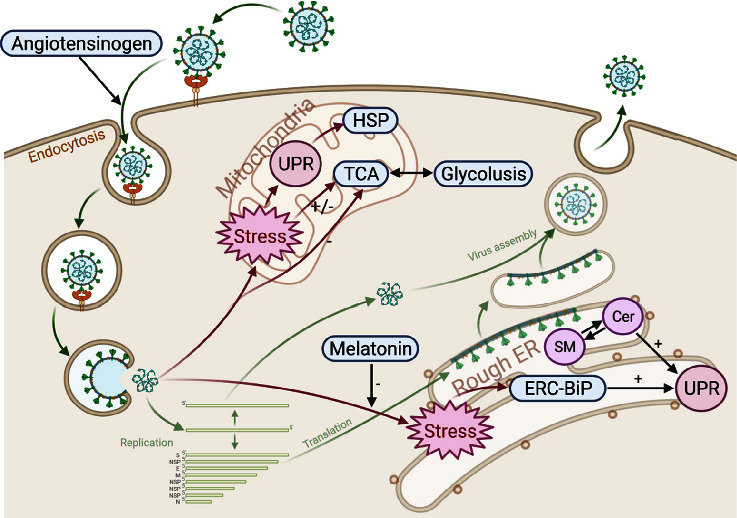
SARS-CoV-2 invades cells and induces endoplasmic reticulum and mitochondrial stress mechanisms. ERC-BiP: endoplasmic reticulum chaperonin-BiP; HSP: heat shock protein; UPR: unfolded protein response; TCA: tricarboxylic acid cycle; SM: sphingomyelin; Cer: ceramide acid; ER: endoplasmic reticulum.

In this study, a functional protein network was established to assess the ER stress state (Figure 4). ERC-BiP was selected as the key pathway protein to evaluate the ER function. Upstream AGT is the key protein essential for the entry of SARS-CoV-2, while downstream melatonin is the ER stress-inhibiting protein. The two act as “valves” regulating pathway activation, thus controlling UPR intensity. The imbalance of Cer-SM, which is closely associated with membrane function, further indicates that ER dysfunction occurs in COVID-19 patients. TCA circulation is inhibited in COVID-19 patients due to hypoxia and mitochondrial dysfunction. The accumulation of lactic acid further destroys the ER protein synthesis system and activates the pyroptosis effect. Both virus and inflammation eventually result in cytokine release syndrome (CRS), which is the leading cause of multiple organ damage and death in severe COVID-19 patients [34]. Over-release of cytokines, injury of respiratory epithelial cells, and accumulation of immune cells lead to a vicious cycle of acute lung injury (ALI)/acute respiratory distress syndrome (ARDS) [6].

### 4.5. Relationship between ERC-BiP and HSP70

BiP is a molecular chaperone of HSP70 located within the endoplasmic reticulum (ER) cavity, which binds to newly synthesized proteins when they are translocated into the ER and maintains them in a state suitable for subsequent folding and oligomerization. BiP is also an important part of the translocation mechanism and plays a role in retrograde transport of abnormal proteins through the ER (which is eventually degraded by the proteasome). BiP is an abundant protein under all growth conditions, but its synthesis is significantly induced under conditions that lead to the accumulation of unfolded peptides in ER [35]. As an ER molecular chaperone, BiP is also required to introduce peptides into the ER cavity or ER membrane in an ATP-dependent manner. ATPase mutants of BiP have been found to block translocation of many proteins (sucrase, carboxypeptidase Y, and factor a) into the endoplasmic network cavity [36–38]. However, there are no studies to explore the relationship between HSP70 and ERC-BiP in the plasma of COVID-19 patients. Although this study accurately detected the value of ERC-BiP, demonstrating a linear correlation between ERC-BiP and disease progression of COVID-19, the detection of HSP70 may provide another new diagnostic marker for the disease, which needs further research in the future.

## 5. Conclusion

The ER plays an important role in the activation of inflammation and innate immunity since it is the main organelle for SARS-CoV-2 assembly. Therefore, the ER functional correlation pathway analysis is crucial for evaluating the mechanism of COVID-19 progression. In this study, ERC-BiP was used as the central protein of the pathway to evaluate the ER function. ERC-BiP was linearly correlated with disease progression. A functional pathway of AGT/ERC-BiP/glycolysis combined with the operation mode of intracellular metabolic pathways was established to evaluate the stress state of intracellular ER after SARS-CoV-2 invasion. Therefore, this study may provide insights into the mechanism of COVID-19 disease progression.

## Figures and Tables

**Table 1:** Basic information of healthy controls and COVID-19 patients.

	Healthy controls	COVID-19	*P*
*N*	17	46	—
Age	34.00 (28.00, 45.50)	46.50 (31.25, 66.00)	0.083
PCT, ng/ml	—	0.10 (0.05, 0.26)	—
SAA, ng/ml	—	85.311 (17.88, 231.90)	—
K, mmol/L	—	3.78 (3.50, 4.67)	—
Na, mmol/L	—	139.00 (136.90, 142.00)	—
Ca, mmol/L	—	2.25 (2.13, 2.32)	—
IL-6, pg/ml	2.01 (0.91, 3.02)	34.73 (28.39, 66.01)	0.001
IL-10, pg/ml	0.59 (0.32, 4.75)	8.36 (4.75, 9.27)	0.001

Blood cell detection			
WBC, 10^9^/L	6.36 (5.41, 6.81)	5.80 (4.66, 8.07)	0.933
NEU, 10^9^/L	3.59 (3.15, 4.32)	3.69 (2.66, 5.60)	0.565
NEU%	60.20 (53.70, 62.50)	64.50 (54.90, 74.15)	0.058
LYM, 10^9^/L	1.84 (1.65, 2.43)	1.35 (0.99, 1.79)	0.001
LYM%	32.50 (28.00, 38.40)	23.90 (14.25, 33.60)	0.002
MONO, 10^9^/L	0.33 (0.25, 0.37)	0.44 (0.33, 0.58)	0.001
MONO%	5.30 (4.30, 5.60)	7.50 (5.60, 10.00)	0.001
BASO, 10^9^/L	0.04 (0.02, 0.05)	0.02 (0.01, 0.03)	0.001
BASO%	0.70 (0.40, 0.80)	0.30 (0.20, 0.50)	0.001
EOS, 10^9^/L	0.13 (0.06, 0.22)	0.09 (0.01, 0.20)	0.196
EOS%	1.10 (2.10, 3.30)	1.50 (0.20, 3.40)	0.180

Coagulation tests			
APTT, second	—	36.00 (30.46, 40.00)	—
PT, second	—	13.30 (12.62, 14.20)	—
INR	—	1.09 (1.02, 1.15)	—
D-dimer, *μ*g/L	—	2.04 (0.74, 1050.00)	—
FIB, g/L	—	3.43 (2.80, 4.57)	—

CRP: C-reactive protein; PCT: procalcitonin; SAA: human serum amyloid A; WBC: white blood cell; NEU: neutrophil; LYM: lymphocyte; MONO: monocyte; BASO: basophil; EOS: eosinophils; APTT: activated partial thromboplastin time; PT: prothrombin time; INR: international normalized ratio; FIB: fibrinogen.

**Table 2:** Comparative analysis of protein and metabolites between healthy controls and COVID-19 patients.

Metabolites	Patient/health				Severe/mild				Total
	log2(FC)	*P*	FDR	VIP	log2(FC)	*P*	FDR	VIP	*P*
ERC-BiP	0.615	0.035	0.003	0.690	0.995	0.018	0.014	1.577	0.019
Lactic acid	−0.946	<0.001	<0.001	1.768	0.167	0.191	0.066	0.471	<0.001
Pyruvic acid	−0.361	0.002	<0.001	0.952	0.156	0.116	0.053	0.412	0.003
Cholesterol	−0.300	0.166	0.014	0.821	0.018	0.400	0.095	0.252	0.263
Melatonin	0.486	<0.001	<0.001	1.098	0.132	0.680	0.152	0.424	0.001
CRP	1.191	0.096	0.008	0.481	2.336	0.002	0.003	1.611	0.004
AGT	2.002	<0.001	<0.001	0.546	2.620	0.000	<0.001	1.859	<0.001
Cer	1.712	<0.001	<0.001	0.967	−0.004	0.353	0.085	0.255	<0.001
SM	−0.947	<0.001	<0.001	1.367	−1.148	0.004	0.004	1.189	<0.001
Oxaloacetic acid	−0.668	0.021	0.002	0.822	0.701	0.070	0.040	0.926	0.009
Malic acid	−0.314	0.017	0.002	0.856	−0.199	0.258	0.075	0.678	0.033
*α*-ketoglutaric acid	−0.333	0.008	0.001	0.870	−0.369	0.353	0.085	0.960	0.019
Isocitrate acid	0.742	<0.001	<0.001	1.160	−0.327	0.123	0.055	0.767	<0.001
Succinic acid	−0.346	0.012	0.001	0.877	0.362	0.191	0.066	0.721	0.016

**Table 3:** Comparative analysis of the temporal fold changes of protein and metabolite between patients in aggravation group and remission group.

Metabolites	Aggravation	Remission	*P*
*n*	29	12	
ERC-BiP	1.66 (1.15, 2.22)	0.88 (0.74, 1.03)	0.001
Lactic acid	1.15 (0.89, 1.47)	1.04 (0.96, 1.26)	0.796
Pyruvic acid	1.02 (0.93, 1.26)	0.99 (0.92, 1.12)	0.456
Cholesterol	1.01 (0.96, 1.11)	1.03 (0.98, 1.07)	0.864
Melatonin	0.99 (0.80, 1.12)	0.83 (0.80, 1.25)	0.528
CRP	1.36 (0.76, 1.95)	0.52 (0.37, 0.85)	0.025
AGT	1.46 (1.20, 2.20)	0.80 (0.59, 0.98)	<0.001
Cer	1.50 (1.00, 2.47)	0.73 (0.50, 1.09)	0.001
SM	0.69 (0.34, 0.86)	1.04 (1.00, 1.49)	0.001
Oxaloacetic acid	1.24 (0.78, 1.69)	0.97 (0.73, 1.06)	0.187
Malic acid	0.77 (0.44, 0.93)	0.95 (0.85, 1.26)	0.071
*α*-ketoglutaric acid	0.97 (0.59, 1.48)	1.43 (0.96, 1.95)	0.136
Isocitrate acid	1.32 (0.85, 1.85)	1.17 (0.98, 1.59)	0.931
Succinic acid	0.92 (0.61, 1.32)	0.90 (0.63, 1.56)	0.864

## Data Availability

The data that support the findings of this study are available from the corresponding authors.

## Supplementary Materials

Supplementary Materials Supplementary table 1: the basic information of patients with disease aggravation and remission. Supplementary table 2: the detailed values of correlation coefficients and *P* values. Supplementary figure 1: the PLS-DA score plot of COVID-19 patients vs. healthy controls and severe patients vs. mild patients.
