# Supplementary material for: ERC-BiP Functional Protein Pathway for Assessing Endoplasmic Reticulum Stress Induced by SARS-CoV-2 Replication after Cell Invasion
Source: Can J Infect Dis Med Microbiol. 2023 Oct 9;2023:7253779. doi: 10.1155/2023/7253779 (PMC10578982; doi:10.1155/2023/7253779)
Supplement: Supplementary Materials — Supplementary table 1: the basic information of patients with disease aggravation and remission. Supplementary table 2: the detailed values of correlation coefficients and P values. Supplementary figure 1: the PLS-DA score plot of COVID-19 patients vs. healthy controls and severe patients vs. mild patients. [file 7253779.f1.zip › Supplementary Figure 1.docx]

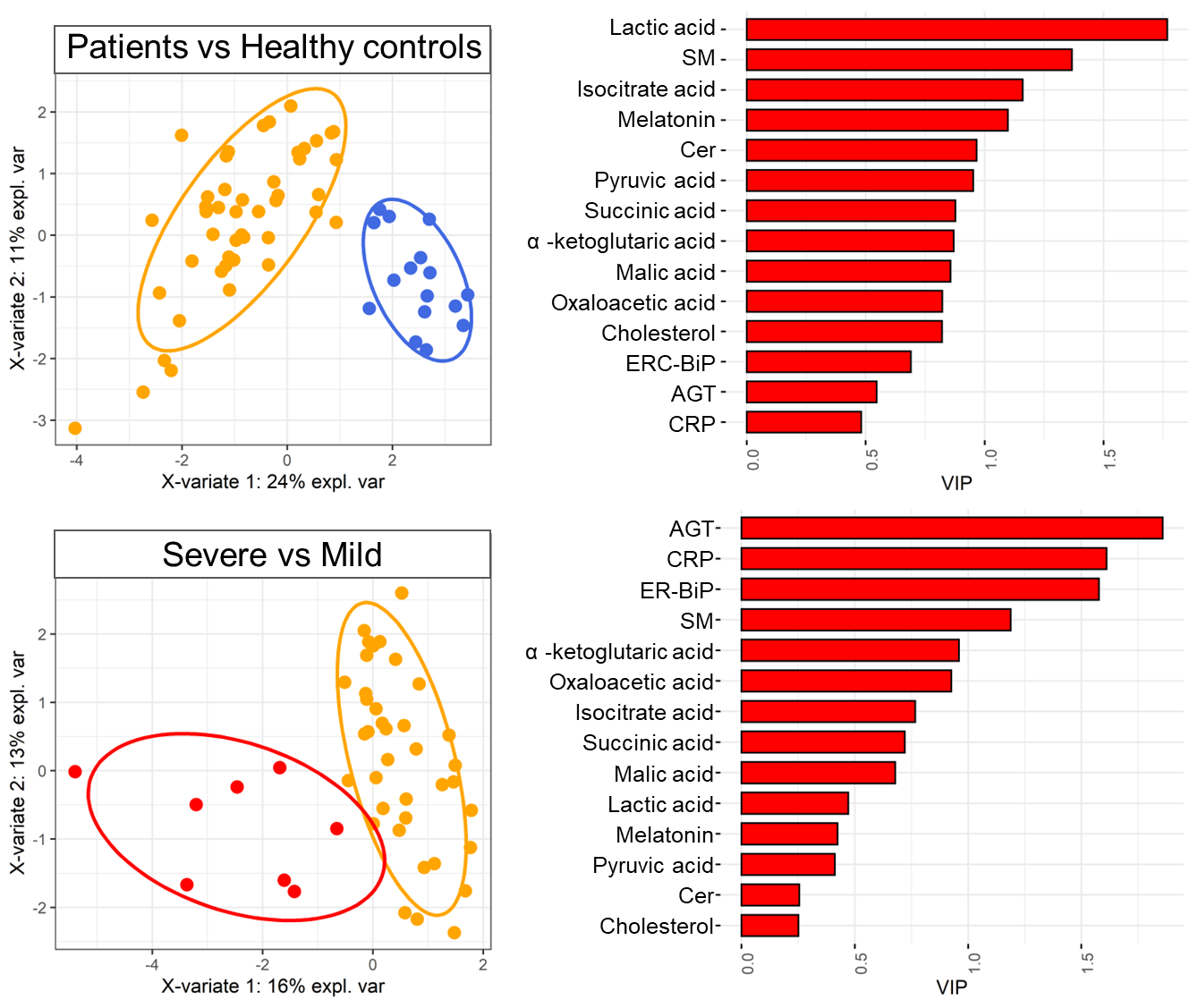


Supplementary figure 1: PLS-DA score plot of COVID-19 patients vs healthy controls and severe patients vs mild patients.
