# Supplementary material for: ERC-BiP Functional Protein Pathway for Assessing Endoplasmic Reticulum Stress Induced by SARS-CoV-2 Replication after Cell Invasion
Source: Can J Infect Dis Med Microbiol. 2023 Oct 9;2023:7253779. doi: 10.1155/2023/7253779 (PMC10578982; doi:10.1155/2023/7253779)
Supplement: Supplementary Materials — Supplementary table 1: the basic information of patients with disease aggravation and remission. Supplementary table 2: the detailed values of correlation coefficients and P values. Supplementary figure 1: the PLS-DA score plot of COVID-19 patients vs. healthy controls and severe patients vs. mild patients. [file 7253779.f1.zip › Supplementary Table 1.docx]

|  | Aggravation | Remission | *P* |
| --- | --- | --- | --- |
| N | 29 | 12 | —— |
| Age | 45.00 [33.00, 62.00] | 56.50 [34.25, 70.50] | 0.439 |
| Male | 14 (48.3) | 7 (41.2) | 0.873 |
| PCT, ng/ml | 0.04 [0.02, 0.07] | 0.03 [0.03, 0.10] | 0.654 |
| SAA, ng/ml | 14.15 [8.21, 37.90] | 40.32 [12.86, 63.37] | 0.457 |
| K, mmol/L | 3.60 [3.40, 3.84] | 3.54 [3.32, 3.90] | 0.938 |
| Na, mmol/L | 139.00 [138.00, 141.20] | 138.75 [136.50, 140.95] | 0.659 |
| Ca, mmol/L | 2.25 [2.20, 2.31] | —— | —— |
| ***Blood cell detection*** |  |  |  |
| WBC, 10^9/L | 5.08 [4.13, 5.93] | 5.03 [4.57, 5.82] | 0.896 |
| NEU, 10^9/L | 3.26 [2.17, 4.30] | 3.71 [2.80, 3.99] | 0.618 |
| NEU% | 66.20 [46.20, 73.50] | 72.05 [61.65, 75.15] | 0.192 |
| LYM, 10^9/L | 1.13 [1.03, 1.64] | 0.98 [0.86, 1.20] | 0.068 |
| LYM% | 23.90 [17.10, 39.40] | 19.00 [15.68, 24.23] | 0.308 |
| MONO, 10^9/L | 0.44 [0.35, 0.62] | 0.44 [0.32, 0.50] | 0.386 |
| MONO% | 9.80 [7.70, 12.60] | 9.40 [5.53, 10.45] | 0.286 |
| BASO, 10^9/L | 0.01 [0.01, 0.02] | 0.01 [0.01, 0.02] | 0.99 |
| BASO% | 0.30 [0.20, 0.40] | 0.30 [0.20, 0.50] | 0.745 |
| EOS, 10^9/L | 0.02 [0.01, 0.05] | 0.01 [0.00, 0.03] | 0.092 |
| EOS% | 0.40 [0.20, 1.30] | 0.15 [0.00, 0.52] | 0.092 |
| ***Coagulation tests*** |  |  |  |
| APTT, second | 35.60 [29.04, 39.10] | 36.10 [31.40, 42.50] | 0.522 |
| PT, second | 13.50 [12.92, 14.01] | 13.26 [13.10, 13.60] | 0.391 |
| INR | 1.07 [1.02, 1.14] | 1.03 [0.99, 1.10] | 0.086 |
| D-Dimer, ug/L | 500.00 [0.35, 1220.00] | 780.00 [0.44, 1340.00] | 0.422 |
| FIB, g/L | 3.04 [2.78, 3.93] | 3.01 [2.68, 3.60] | 0.605 |
| ***Metabolites and Proteins*** |  |  |  |
| ERC-BiP | 139102.00 [98270.10, 206168.00] | 183696.00 [125609.00, 228933.00] | 0.25 |
| Lactic acid | 187130000.00 [153670000.00, 223480000.00] | 190060000.00 [165360000.00, 219720000.00] | 0.741 |
| Pyruvic acid | 9775900.00 [8161600.00, 11409000.00] | 10058000.00 [9279900.00, 11794000.00] | 0.432 |
| Cholesterol | 4702700.00 [4291600.00, 4983800.00] | 4777300.00 [4531100.00, 4896300.00] | 0.741 |
| Melatonin | 101000.00 [87204.00, 118010.00] | 111420.00 [90315.00, 120010.00] | 0.432 |
| CRP | 261909.00 [109047.00, 438229.00] | 146464.00 [74839.00, 387160.00] | 0.345 |
| AGT | 541156.00 [310809.00, 1091730.00] | 273108.00 [134912.00, 354516.00] | 0.032 |
| Cer | 17554.00 [12711.00, 31544.00] | 16787.00 [11232.00, 32866.00] | 0.882 |
| SM | 1229500.00 [878740.00, 1816600.00] | 1441100.00 [589910.00, 1736600.00] | 0.82 |
| Oxaloacetic acid | 96328.00 [65567.00, 291280.00] | 77068.00 [61511.00, 137460.00] | 0.419 |
| Malic acid | 274390.00 [229550.00, 340850.00] | 234110.00 [196510.00, 272690.00] | 0.07 |
| α -ketoglutaric acid | 1059100.00 [844120.00, 1396600.00] | 925550.00 [666450.00, 1443100.00] | 0.488 |
| Isocitrate acid | 83039.00 [60927.00, 93885.00] | 60441.00 [48246.00, 92650.00] | 0.473 |
| Succinic acid | 1968700.00 [1632600.00, 2490700.00] | 1883500.00 [1769400.00, 2247400.00] | 0.882 |
| TCA-Cycle | 3828446.00 [3439074.00, 4616201.00] | 3778663.00 [3259556.00, 4515438.00] | 0.577 |
