# Supplementary material for: ERC-BiP Functional Protein Pathway for Assessing Endoplasmic Reticulum Stress Induced by SARS-CoV-2 Replication after Cell Invasion
Source: Can J Infect Dis Med Microbiol. 2023 Oct 9;2023:7253779. doi: 10.1155/2023/7253779 (PMC10578982; doi:10.1155/2023/7253779)
Supplement: Supplementary Materials — Supplementary table 1: the basic information of patients with disease aggravation and remission. Supplementary table 2: the detailed values of correlation coefficients and P values. Supplementary figure 1: the PLS-DA score plot of COVID-19 patients vs. healthy controls and severe patients vs. mild patients. [file 7253779.f1.zip › Supplementary Table 2.docx]

|  | WBC | NEU | NEU% | LYM | LYM% | MONO | MONO% | SAA | PCT | IL-6 | IL-10 | ERC-BiP | Lactic acid | Pyruvic acid | Cholesterol | Melatonin | CRP | AGT | Cer | SM | Oxaloacetic acid | Malic acid | α -ketoglutaric acid | Isocitrate acid | Succinic acid | TCA-Cycle |
| --- | --- | --- | --- | --- | --- | --- | --- | --- | --- | --- | --- | --- | --- | --- | --- | --- | --- | --- | --- | --- | --- | --- | --- | --- | --- | --- |
| WBC | 1.0000 | 0.8765 | 0.4033 | 0.1430 | -0.3963 | 0.3445 | -0.1948 | -0.2104 | 0.3817 | 0.4248 | 0.1624 | -0.1664 | 0.2487 | 0.3046 | 0.1223 | -0.2513 | -0.2089 | -0.1636 | -0.1784 | -0.0489 | 0.4937 | 0.1337 | 0.0825 | -0.2652 | -0.0306 | 0.0914 |
| NEU | 0.0000 | 1.0000 | 0.7530 | -0.2761 | -0.7249 | 0.3632 | -0.1499 | -0.0403 | 0.4968 | 0.4742 | 0.2356 | -0.2287 | 0.0835 | 0.2747 | 0.1077 | -0.1434 | -0.1327 | -0.0651 | -0.0134 | -0.2305 | 0.5349 | -0.0211 | -0.0097 | -0.2463 | -0.1587 | -0.0470 |
| NEU% | 0.0012 | 0.0000 | 1.0000 | -0.7485 | -0.9476 | 0.2399 | -0.0691 | 0.2338 | 0.5890 | 0.4158 | 0.3823 | -0.1542 | -0.0865 | 0.1947 | -0.0271 | 0.1103 | 0.0386 | 0.1276 | 0.2850 | -0.4083 | 0.3922 | -0.1375 | -0.0896 | -0.1251 | -0.2336 | -0.1218 |
| LYM | 0.2675 | 0.0298 | 0.0000 | 1.0000 | 0.8235 | -0.3047 | -0.3610 | -0.3314 | -0.4715 | -0.3274 | -0.5958 | 0.0485 | 0.3469 | -0.0200 | 0.1163 | -0.2540 | -0.0192 | -0.1824 | -0.3465 | 0.4272 | -0.1493 | 0.1633 | 0.0702 | -0.1033 | 0.3111 | 0.2258 |
| LYM% | 0.0014 | 0.0000 | 0.0000 | 0.0000 | 1.0000 | -0.4399 | -0.1754 | -0.2209 | -0.5556 | -0.3662 | -0.4701 | 0.1346 | 0.1689 | -0.1959 | 0.0489 | -0.1334 | 0.0148 | -0.1448 | -0.2563 | 0.4681 | -0.3809 | 0.1008 | 0.0620 | 0.0626 | 0.2863 | 0.1619 |
| MONO | 0.0061 | 0.0037 | 0.0604 | 0.0160 | 0.0003 | 1.0000 | 0.7792 | -0.1728 | 0.2366 | 0.2643 | 0.2941 | -0.1104 | -0.3715 | 0.0738 | -0.0431 | 0.0748 | -0.0186 | 0.0532 | 0.0067 | -0.3663 | 0.1961 | 0.0156 | -0.0107 | -0.0019 | -0.4078 | -0.3134 |
| MONO% | 0.1291 | 0.2448 | 0.5938 | 0.0039 | 0.1726 | 0.0000 | 1.0000 | -0.2377 | 0.0304 | 0.0284 | 0.2179 | -0.0076 | -0.5147 | -0.1559 | -0.0734 | 0.1098 | 0.0042 | 0.0754 | 0.0394 | -0.3313 | -0.0517 | 0.0329 | 0.0081 | 0.2222 | -0.3690 | -0.3238 |
| SAA | 0.1007 | 0.7560 | 0.0675 | 0.0085 | 0.0844 | 0.1791 | 0.0628 | 1.0000 | 0.1506 | 0.3707 | 0.4502 | -0.2052 | -0.0429 | -0.1468 | 0.6403 | -0.0104 | 0.0403 | -0.0494 | -0.0519 | -0.6948 | 0.0403 | -0.2636 | -0.3078 | 0.0792 | -0.0273 | -0.0299 |
| PCT | 0.0022 | 0.0000 | 0.0000 | 0.0001 | 0.0000 | 0.0641 | 0.8145 | 0.2426 | 1.0000 | 0.7603 | 0.5372 | -0.1849 | -0.1701 | 0.2032 | -0.2176 | -0.4193 | 0.0361 | 0.1958 | 0.3417 | -0.5632 | 0.7575 | 0.2423 | 0.0440 | -0.5998 | 0.2670 | 0.4944 |
| IL-6 | 0.0006 | 0.0001 | 0.0008 | 0.0094 | 0.0034 | 0.0379 | 0.8265 | 0.0030 | 0.0000 | 1.0000 | 0.6953 | -0.3004 | 0.0043 | 0.4088 | 0.1701 | 0.1478 | 0.0165 | -0.1813 | -0.0837 | -0.1600 | 0.6747 | 0.1117 | 0.2085 | -0.4372 | -0.3734 | -0.0724 |
| IL-10 | 0.2071 | 0.0652 | 0.0022 | 0.0000 | 0.0001 | 0.0203 | 0.0889 | 0.0002 | 0.0000 | 0.0000 | 1.0000 | -0.2787 | 0.0403 | 0.3428 | 0.1566 | 0.1310 | 0.0376 | -0.1081 | -0.0559 | -0.3521 | 0.7055 | 0.0742 | 0.0873 | -0.4314 | -0.4799 | -0.2757 |
| ERC-BiP | 0.1960 | 0.0738 | 0.2313 | 0.7079 | 0.2970 | 0.3929 | 0.9530 | 0.1096 | 0.1502 | 0.0177 | 0.0283 | 1.0000 | 0.3529 | -0.0700 | -0.0259 | 0.0774 | -0.0388 | 0.4397 | 0.5341 | 0.0695 | -0.1563 | -0.3565 | -0.0985 | 0.0846 | -0.0899 | -0.2285 |
| Lactic acid | 0.0513 | 0.5189 | 0.5039 | 0.0057 | 0.1895 | 0.0029 | 0.0000 | 0.7408 | 0.1863 | 0.9737 | 0.7558 | 0.0049 | 1.0000 | 0.4828 | 0.2340 | -0.2327 | -0.2019 | -0.2616 | -0.1759 | 0.4875 | 0.2388 | 0.0603 | 0.2309 | -0.3347 | 0.1683 | 0.1981 |
| Pyruvic acid | 0.0161 | 0.0307 | 0.1294 | 0.8773 | 0.1270 | 0.5687 | 0.2262 | 0.2550 | 0.1132 | 0.0010 | 0.0064 | 0.5889 | 0.0001 | 1.0000 | 0.1415 | 0.1066 | -0.2157 | -0.2470 | -0.2310 | 0.3381 | 0.3760 | 0.2743 | 0.3684 | -0.2978 | 0.0376 | 0.1755 |
| Cholesterol | 0.3438 | 0.4048 | 0.8341 | 0.3680 | 0.7058 | 0.7394 | 0.5708 | 0.0000 | 0.0894 | 0.1864 | 0.2241 | 0.8415 | 0.0672 | 0.2725 | 1.0000 | -0.0686 | -0.1614 | -0.1361 | -0.2256 | -0.0065 | 0.0716 | -0.1743 | 0.1380 | 0.0065 | -0.0738 | -0.0218 |
| Melatonin | 0.0488 | 0.2660 | 0.3932 | 0.0464 | 0.3014 | 0.5632 | 0.3956 | 0.9361 | 0.0007 | 0.2517 | 0.3103 | 0.5497 | 0.0688 | 0.4094 | 0.5964 | 1.0000 | 0.2324 | 0.2703 | 0.2949 | -0.0677 | -0.1862 | -0.3348 | -0.1872 | 0.3086 | -0.1573 | -0.2339 |
| CRP | 0.1032 | 0.3039 | 0.7660 | 0.8821 | 0.9092 | 0.8859 | 0.9742 | 0.7560 | 0.7806 | 0.8988 | 0.7720 | 0.7646 | 0.1156 | 0.0922 | 0.2101 | 0.0691 | 1.0000 | 0.3436 | 0.2752 | -0.2830 | -0.0528 | -0.1898 | -0.2271 | -0.0220 | 0.0448 | -0.0567 |
| AGT | 0.2040 | 0.6154 | 0.3231 | 0.1559 | 0.2616 | 0.6813 | 0.5603 | 0.7033 | 0.1272 | 0.1584 | 0.4031 | 0.0003 | 0.0400 | 0.0529 | 0.2915 | 0.0336 | 0.0063 | 1.0000 | 0.6922 | -0.5144 | -0.1141 | -0.2757 | -0.3814 | 0.1275 | 0.0741 | -0.1016 |
| Cer | 0.1654 | 0.9175 | 0.0248 | 0.0058 | 0.0444 | 0.9586 | 0.7611 | 0.6884 | 0.0066 | 0.5180 | 0.6663 | 0.0000 | 0.1715 | 0.0708 | 0.0780 | 0.0200 | 0.0304 | 0.0000 | 1.0000 | -0.3157 | -0.0727 | -0.2726 | -0.2439 | 0.1138 | -0.0407 | -0.1283 |
| SM | 0.7057 | 0.0715 | 0.0010 | 0.0005 | 0.0001 | 0.0034 | 0.0085 | 0.0000 | 0.0000 | 0.2142 | 0.0050 | 0.5913 | 0.0001 | 0.0072 | 0.9597 | 0.6010 | 0.0258 | 0.0000 | 0.0124 | 1.0000 | -0.0314 | 0.2265 | 0.5543 | -0.0223 | 0.0571 | 0.2311 |
| Oxaloacetic acid | 0.0000 | 0.0000 | 0.0016 | 0.2467 | 0.0023 | 0.1267 | 0.6897 | 0.7560 | 0.0000 | 0.0000 | 0.0000 | 0.2251 | 0.0616 | 0.0026 | 0.5800 | 0.1474 | 0.6836 | 0.3770 | 0.5744 | 0.8084 | 1.0000 | 0.3204 | 0.3560 | -0.3677 | -0.1162 | 0.1856 |
| Malic acid | 0.3002 | 0.8707 | 0.2866 | 0.2048 | 0.4355 | 0.9040 | 0.7998 | 0.0384 | 0.0578 | 0.3872 | 0.5666 | 0.0045 | 0.6415 | 0.0310 | 0.1754 | 0.0078 | 0.1395 | 0.0301 | 0.0320 | 0.0767 | 0.0111 | 1.0000 | 0.5978 | -0.0114 | 0.2647 | 0.6404 |
| α -ketoglutaric acid | 0.5237 | 0.9405 | 0.4887 | 0.5877 | 0.6323 | 0.9340 | 0.9499 | 0.0149 | 0.7342 | 0.1039 | 0.4998 | 0.4463 | 0.0710 | 0.0032 | 0.2849 | 0.1451 | 0.0759 | 0.0022 | 0.0560 | 0.0000 | 0.0045 | 0.0000 | 1.0000 | 0.2077 | -0.0616 | 0.5016 |
| Isocitrate acid | 0.0372 | 0.0537 | 0.3328 | 0.4245 | 0.6287 | 0.9885 | 0.0826 | 0.5405 | 0.0000 | 0.0004 | 0.0005 | 0.5133 | 0.0078 | 0.0187 | 0.9599 | 0.0147 | 0.8653 | 0.3232 | 0.3783 | 0.8633 | 0.0033 | 0.9302 | 0.1053 | 1.0000 | -0.1438 | 0.0092 |
| Succinic acid | 0.8134 | 0.2180 | 0.0677 | 0.0138 | 0.0241 | 0.0010 | 0.0032 | 0.8333 | 0.0359 | 0.0028 | 0.0001 | 0.4870 | 0.1910 | 0.7714 | 0.5686 | 0.2221 | 0.7292 | 0.5670 | 0.7533 | 0.6593 | 0.3686 | 0.0376 | 0.6342 | 0.2648 | 1.0000 | 0.7544 |
| TCA-Cycle | 0.4796 | 0.7165 | 0.3457 | 0.0777 | 0.2086 | 0.0131 | 0.0102 | 0.8177 | 0.0000 | 0.5763 | 0.0301 | 0.0740 | 0.1227 | 0.1723 | 0.8662 | 0.0673 | 0.6617 | 0.4322 | 0.3205 | 0.0707 | 0.1486 | 0.0000 | 0.0000 | 0.9432 | 0.0000 | 1.0000 |
